# Ultrasound-Guided Hip Injections with High Density Hyaluronic Acid: Outcome at One Year Follow Up

**DOI:** 10.3390/jcm13092515

**Published:** 2024-04-25

**Authors:** Antonino Giulio Battaglia, Rocco D’Apolito, Fabio Labionda, Joil Ramazzotti, Luigi Zagra

**Affiliations:** IRCCS Istituto Ortopedico Galeazzi, Hip Department, 20157 Milan, Italy; roccodapolito@hotmail.it (R.D.); f.sklab@gmail.com (F.L.); joil.ramazzotti3449@gmail.com (J.R.); luigi.zagra@fastwebnet.it (L.Z.)

**Keywords:** hip, intra-articular injection, hyaluronic acid, hip preservation, osteoarthritis, viscosupplementation

## Abstract

**Background**: The ultrasound-guided viscosupplementation of the hip joint with hyaluronic acid (HA) is considered a standard procedure among the conservative treatments for hip arthritis. The aim of this study was to evaluate the clinical benefit and the incidence of adverse events of the technique in an observational study at one year follow up. **Methods:** We evaluated a consecutive series of 85 patients with a diagnosis of symptomatic arthritis who underwent intra-articular ultrasound-guided hyaluronic acid injections. The scales used for evaluation were modified Harris Hip Score (mHHS), WOMAC (Western Ontario and McMaster University), and Hip Outcome Score (HOS) with subscale Sport (HOSs), for pain the Visual Analogic Scale (VAS). The patients were classified according to Tonnis’ radiological classification of arthritis (range 0–3): 20 patients (grade 0), 32 (grade 1), 18 (grade 2), 15 (grade 3). **Results:** At last follow up, all the scales increased: mHHS from 59.35 to 82.1, HOS from 69.45 to 78.53, HOss from 47.4 to 58.11, VAS from 6.09 to 3.97, WOMAC from 33.2 to 31.5 (*p* < 0.05 for all the parameters); the results were elaborated with GraphPad Prism v5.0 (Prism Software La Jolla, CA, USA) using Wilcoxon’s test. A total of 13 patients out of 85 needed arthroplasty, all classified as Tonnis grade 3. No serious adverse events were noted due to the procedure. **Conclusions:** Based on our findings, indication for the use of hyaluronic acid is limited to patients with mild to moderate arthritis. Patients in advanced arthritis refusing replacement surgery and asking for this treatment should be informed about the poor results of the technique even in the short term.

## 1. Introduction

Osteoarthritis (OA) is a common degenerative disorder of the articular cartilage associated with hypertrophic bone changes. Risk factors include genetics, female sex, past trauma, advancing age, and obesity [1]. The diagnosis of OA is typically based on a history of joint pain that worsens with movement, which can lead to disability in activities of daily living. It is worth noting that although OA is more commonly found in elderly individuals, symptomatic hip OA can still be found in middle-aged patients [2]. It represents one of the leading causes of hip pain in adults and has one of the highest financial burdens [3]. Among adults 45 years of age, the prevalence ranges from 6.7% to 9.2% [4], increasing to 25% in patients aged over 55 years, constituting a reason for chronic joint pain and stiffness [5]. The diagnosis of hip pain can be difficult because the discomfort can originate from different locations and in consideration of compensatory pain patterns. Pain generators can include the intra- and extra-articular hip structures, the lumbar spine, the pelvic floor, or a combination of these. Hip pain can also be caused by issues related to the bowel, bladder, or reproductive organs. Optimal management of OA requires a combination of non-pharmacological and pharmacological modalities.

Injections in and around the hip have become a significant aspect of both diagnostic and nonsurgical treatment methods for hip pain. Advantages of hip injections include being easy to perform, minimally invasive, fast, and relatively inexpensive. The fact that the hip is deeper than the other joints, the proximity to important neurovascular structures, and lack of palpable anatomic landmarks can make it difficult to judge the position of the tip of the needle prior to the injection and consequently the correct execution of the procedure. Considering the available options, ultrasound could be considered as the preferred method. Actually, ultrasound has some limitations in its ability to penetrate bony structures, but it is highly effective in visualizing soft tissues and vascular structures in areas of anatomical interest and has become more and more used for posterior neuraxial, periaxial, peripheral nerve, and joint-related structures. By observing the patient’s response to these injections, it is possible to better select patients for surgery and provide improved pain control for the soft-tissue pathology that often accompanies intra-articular pathology. With regard to injections, ultrasound-guided injections (UGIs) are significantly more accurate than those performed without guidance (70–88% vs. 97%) [6]. Ultrasound guidance has proved superiority at accuracy of delivery and procedural effectiveness over blind procedures when used in association with interventional pain procedures [7]. Moreover, compared to fluoroscopic guidance, ultrasound has the advantage to be without radiation exposure both for the patient and the physician, which caused increased popularity of this method in the orthopedic community over the fluoroscopy, alongside the chance of having real-time visualization of dynamic anatomy.

Intraarticular hip injections have been used for many years [8]. Crowe [9] reported good results with intraarticular acid phosphate injections for the treatment of hip OA. The anterior approach was recommended for administration of the injection as the easiest, least painful, and most accurate. Some years later, Leveaux and Quin [10] published their results on the combination of hydrocortisone and procaine for the treatment of osteoarthritis of the hip and concluded that the combination of these two substances was a valuable palliative management for the painful osteoarthritic hip joint. Currently, different medications can be used for hip injections (HI), including corticosteroids, hyaluronic acid (HA), platelet-rich-plasma (PRP), mesenchymal stem cells (MSCs), and many different combinations, although the true extent of their benefits is still debated [11]. The injection of corticosteroids (generally, 1 mL) has been invoked in literature for early hip OA [12] and determines short-to-mid-term response, whereas the efficacy of HA seems to be longer, similar to that of platelet-rich-plasma [13]. However, debated data have been reported about the actual superiority of HA to corticosteroids or placebo [14] and the American Academy of Orthopedic Surgeons (AAOS) guidelines on the management of OA of the hip published in 2023 [15] considered only intraarticular CSs and HA worthy of any recommendation. AAOS supported the use of intraarticular CSs to improve short-term function and pain for patients with symptomatic OA of the hip. However, they did not support the use of intraarticular HA, citing equal efficacy to placebo for function, stiffness, and pain in patients with symptomatic OA of the hip. HA was first isolated in 1934 by Karl Meyer in the vitreous humor; the first human clinical use of intra-articular HA in the treatment of knee arthritis was made in 1975, and the clinical studies were published during the eighties [16]. HA is a polysaccharide macromolecule, a glycosaminoglycan of high molecular weight (MW) composed of repetitions of disaccharides of glucuronic acid and *N*-acetylglucosamine; it is part of synovial fluid in normal and osteoarthritic joints and is produced by chondrocytes and synoviocytes [17]. HA has complex biological properties that could explain its analgesic effects (anti-inflammatory by inhibiting the formation and release of prostaglandin, immunomodulatory in situ), irrespective of its mechanical action on the joint fluid [18,19]. The concentration of HA in an arthritic joint decreases by 50–67% and includes a reduction in molecular size. Molecular interaction has also been observed, with a consequent decrease in elasticity and viscosity of the synovial fluid [20]. HA injections might play an important role in delaying the need for total hip arthroplasty (THA) [21], which is particularly important in young patients.

The purpose of this study was to assess the safety and efficacy of high-molecular-weight HA in reducing pain and improving function in hip OA in the clinical setting.

## 2. Materials and Methods

The study population included all patients with moderate–severe hip pain due to hip OA evaluated in according to VAS scale and undergoing hip UGI using high-molecular-weight HA in our department during 2021. This was a pure observational study. This procedure is a consolidate therapy, and it is registered at our institute. Inclusion criteria were symptomatic hip OA, regardless of the radiographic severity of OA according to Tonnis classification, with a minimum of 18 years of age and with no upper age limits. Diagnosis was based on presenting symptoms, clinical history, and examination, supported by evidence of hip OA on anteroposterior (AP) weight-bearing pelvis and lateral view of the hip X-rays. Each radiograph had to be no older than 3 months from the time of injection. AP pelvis and lateral oblique views were obtained as detailed below.

AP pelvis: The patient lies supine on the table with their legs extended and their head resting on a pillow. The median sagittal plane is at 90 degrees to the table-top and the anterior superior iliac spines (ASISs) are at equal distance from the table-top. The arms are raised onto the pillow. The legs are slightly internally rotated to bring the necks of femora parallel to the table-top. Gonad protection is applied if appropriate. The beam is centered in the midline, midway between the ASISs and the upper border of the symphysis pubis.

Lateral oblique hip: from the initial AP pelvis position, the patient is rotated laterally through 45 degrees onto the side under examination and supported in this position with foam pads. The knee and hip are flexed and externally rotated to bring the lateral aspect of the thigh in contact with the table-top. The arms are rested on the pillow. Gonad protection is applied. The beam is centered to the femoral pulse.

Exclusion criteria were: patients already scheduled for surgery, any previous surgery on the affected hip, clinical suspicion of local or systemic sepsis or infection, current or previous infection of the affected hip, significant trauma to the affected hip requiring immobilization in the previous 3 months, inability to understand and complete self-report questionnaires written (or spoken) in Italian, hip pain due to other disorders (e.g., trochanteric bursitis, avascular necrosis, pain referred from the spine, infection), inflammatory joint disease (e.g., rheumatoid arthritis, seronegative spondyloarthropathy, ankylosing spondylitis, psoriatic arthritis, reactive arthritis, inflammatory-bowel disease associated inflammatory arthritis), malignancy (where malignancy is suspected to provoke hip pain), bleeding disorders, any history of hypersensitivity to lidocaine hydrochloride or any of its excipients, pregnant or lactating females, any contraindications to the use of 1% lidocaine hydrochloride as complete heart block, hypovolemia, porphyria, polymyalgia rheumatica, or other condition requiring regular oral steroid use. There were no specific inclusion or exclusion criteria regarding race or gender.

A total of 85 patients (51 females and 34 males) treated in 2021 were enrolled in the study. A total of 35 patients were not included because they did not meet inclusion criteria. Mean age was 57 years old (range, 19–87). The mean follow-up was 12 months (range, 8–14 months). According to Tonnis classification, 20 patients had grade 0, 32 grade 1, 18 grade 2, 15 grade 3. See Table 1.

All patients underwent 2 intra-articular UGIs of high-molecular-weight HA (Hyalubrix^®^ 60 (HA > 1500 kDa, Fidia Farmaceutici Spa-Abano Terme (PD), Italy) added with 1% lidocaine hydrochloride, 4 weeks apart each. Before the injections, they completed questionnaires for clinical scores, repeated at 12 months during a phone interview. The questionnaires included modified Harris Hip Score (mHHS) [22,23], Hip Outcome Score (HOS) [24], Hip Outcome Score Sport (HOSs) [25], Visual Analogic Scale (VAS), and Western Ontario and McMaster University Osteoarthritis index (WOMAC) [26] to assess pain, stiffness, and everyday physical function. These scores were used to assess a clinical evaluation as suggested by Hunt et al. and Mujahed et al. [27,28]. The mHHS is a validated hip score, which was derived from the Harris Hip Score after removing the physician-reported range of movement assessment but retaining the pain and functional scores [29,30]. These two scores have been shown to be comparable [30]. If patients still continue to experience pain after injection(s) due to their hip osteoarthritis, they may opt to undergo total hip arthroplasty. The long-term outcome of total hip arthroplasty is very good and the satisfaction rates (92%) are usually superior to other common orthopedic surgeries (e.g., transforaminal lumbar interbody fusion (86%) and hallux valgus correction (77%)) [31].

## 3. Brief Description of US Imaging

US devices send out pulses of vibrations at frequencies beyond the range of human hearing from a flat (linear) or curved (curvilinear) probe [32]. These pulses produce echoes from interfaces between tissues of differing density. These echoes can be used to construct 2D (planar) or 3D images of the echoing interface. Needle advancement is observed either “in-plane” where the needle continually stays in view by maintaining an acute angle to the line of pulsations or “out-of-plane” where the needle tip enters the plane of imaging as it interacts with the target site and maintains a perpendicular angle to the probe. Differences in density between water, fat, and calcified structures allow real-time imaging of anatomical structures. Resolution of adjacent images is a function of the wavelength of the US; at higher frequencies, greater image resolution is observed but at limited depths, whereas at lower frequencies, depth is increased but at the cost of image resolution. Soft tissues may allow additional echoes to be perceived from areas distal to those tissues but dense tissues such as bone will create image “shadows” that limit visualization of those distal structures. It is possible to observe motion of echoing interfaces (like blood cells in vessels) by sensing Doppler shifts in the frequency of the pulse echoes returning to the probe; objects moving away from the probe will echo a slightly lower frequency, and objects moving towards the probe will echo a slightly higher frequency. This ability to detect motion is used to identify vascular structures and is one of the major advantages of using US.

## 4. Procedure

**Figure 1:**
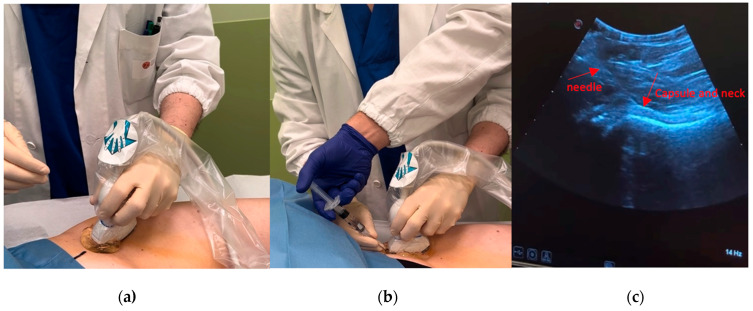
(**a**) Skin cleaned with antiseptic; sterile gel and probe used; (**b**) injection of HA intra-articular with a 20–22 G needle; (**c**) Follow the needle in the monitor to avoid extravasation, observing capsule and hip neck.

UGIs were performed in a dedicated outpatient room by 2 trained orthopedic surgeons (AGB,FL), who are fully trained in the technique and work in the musculoskeletal services supported by a nurse or a resident, with the patient in supine position with legs extended into a neutral position of comfort. The skin is cleaned with 10% Betadine (povidone–iodine) solution. After skin disinfection and delimitation of the field with sterile drapes, probe was prepared. The transducer was covered with gel and a sterile sheath. Sterile gel was applied on the external surface of the sheath on the probe. With the convex probe placed parallel to the femoral neck and the head–neck junction being the target of injection, the needle (a 20–22 G Å~9 cm spinal needle) was inserted with an in-plane cranial-to-caudal approach. The anterolateral approach with caudal–cranial direction of the needle was used rarely, just for overweight patients with a large hip joint. The needle was inserted until it touched bone; if it did not touch bone, the needle was reinserted with a lesser angle to the floor along the same craniocaudal direction until bone was encountered. The beveled needle was rotated while in place to allow more free flow of the injected fluid or slightly withdrawn if resistance continued. Once the needle passed the capsule, the High molecular weight HA (>1500 Kda) was injected. Distension of the capsule confirmed the correct site of injection, and fluid was visualized dispersing through the joint. See Figure 1.

Generally, after the procedure, the patient sat outside the clinic for 10–15 min, holding ice on the injection area before returning home. Participants were advised to wait 15 min following injection or alternatively ensure that they were accompanied by a responsible adult for that time and to observe weight-bearing as tolerated for 24 h following the injection, and not to drive for 24 h. Patients were instructed to perform activities as tolerated with no weight-bearing restrictions after three days.

## 5. Results

The results were elaborated with GraphPad Prism v5.0 (Prism Software La Jolla, CA, USA) using Wilcoxon’s test. Data distribution was assessed by Shapiro–Wilk test, and Wilcoxon matched paired test was used to test the differences between pre- and post-injections score, according to the results of the normality test.

**Figure 2:**
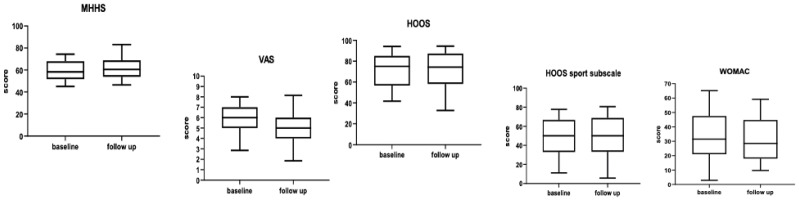
Box and whisker plot showing the distribution of clinical scores at baseline and at 1 year.

After 12 months, mHHS, HOS, HOSs, VAS, and WOMAC values improved from 59.35 to 82.1 (*p* < 0.05), from 69.45 to 78.53 (*p* < 0.05), from 47.4 to 58.11 (*p* < 0.05), VAS from 6.09 to 3.97 (*p* < 0.05), WOMAC from 33.2 to 31.5 (*p* < 0.05); see Figure 2 and Table 2.

We did not observe any adverse effects or infections. A total of 18 patients (21.2%) reported transient post-injection pain from moderate (*n* = 15, 17.7%) to severe (*n* = 3, 3.5%) in the hip joint some hours after procedure that disappeared within 5 days.

**Figure 3:**
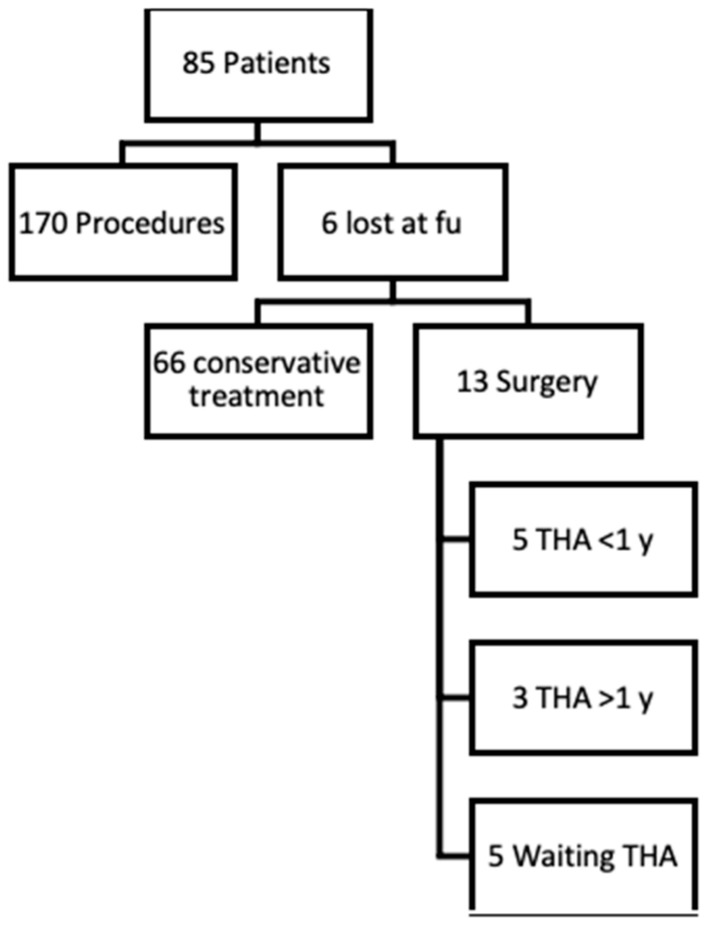
STROBE diagram of hip injections outcomes;8 patients scheduled for THA waited less or more 1 y (Year); 5 are still waiting for surgery.

In the end of the study period, 85 patients met the inclusion criteria. In total, 13 out of 85 (15.3%) patients were scheduled for THA; 5 (5.9%) underwent surgery between 6 and 12 months after UGIs, 3 (3.5%) more than 12 months after, and 5 (5.9%) are still on the waiting list. All 13 patients were classified as Tonnis 3. Six (7%) patients were lost during follow up. A total of 66 out of 85 (77.4%) patients did not require the surgery option at last follow up. See Figure 3.

## 6. Discussion

Our results support UGIs with high-molecular-weight HA as a treatment for the early stages of hip OA such as reported by Kim et al. [33] in selected patients. These authors showed improvements in clinical score in the first months after the injection in younger patients and those affected by mild to moderate arthritis, and negative outcomes in the older and those with more severe Kellgreen–Lawrence grading. More recently, Micu et al. [34], in a case-control study, reported significant improvement in VAS and WOMAC scores up to 6 months after a course of UGIs of HA. Similarly, Migliore et al. [35] showed statistically significant reductions in VAS and drug consumption after 6 and 12 months of follow-up. According to Nouri et al. [36] who studied PRP, HA, and their combination, the combination of the two was more effective in the long run, whereas Ronconi et al. [37] reported good results at 12 months using a combination of HA and corticosteroids.

HA is a major component of synovial fluid and provides lubrication and shock absorption in joints. In OA, there has been shown to be a decrease in HA molecular weight and concentration in the synovial fluid as the disease progresses, leading to a reduction in viscoelastic properties [38]. Hence, there is potential for symptomatic improvement by supplementing HA with better viscoelastic properties. Hyaluronate is composed of alternating N-acetyl-d-glucosamine and d-glucuronic acid residues attached by β(1–4) and β(1–3) bonds with molecular mass ranging from 6500 to 10,900 kDa [18]. Its rheological characteristics involved in the main function of synovial fluid are to serve as a lubricant, scavenger for free radicals, and for the regulation of cellular activities such as binding of proteins [19]. During the progression of OA, the endogenous HA in the joint is depolymerized from being of a high molecular weight (6500–10,900 kDa) into a lower molecular weight (2700–4500 kDa), which consequently diminishes the mechanical and viscoelastic properties of the synovial fluid in the affected joint [28,29]. Thus, exogenous HA injections have been clinically used to mitigate the macerated functions of the depolymerized endogenous HA of OA patients [28]; although the exogenous HA does not restore and replace the full properties and activities of the depolymerized endogenous HA of the synovial fluid, it may induce satisfactory pain relief via several mechanisms [28]. These mechanisms include synthesis of proteoglycan and/or glycosaminoglycan, anti-inflammatory effect, and viscoelasticity maintenance [28]. Nevertheless, there is a clear heterogeneity in the therapeutic trajectory for OA patients following HA injections. Some studies report an overall beneficial effect, while others report that there is only a small benefit [21]. One of the potential reasons for the variable effect of HA treatments on OA patients is levels of hyaluronidases in a patient’s synovial fluid. Hyaluronidases are a family of enzymes that degrade hyaluronic acid through cleaving the β(1–4) linkages of HA, fracturing the large molecule into smaller pieces before degrading it [30]. HA is being administrated into OA patients via two main ways: either oral administration or local injection [31,32]. Several preparations of injectable HA used for clinical use include Synvisc^®^ and Synvisc-One^®^ (Genzyme, Corporation, Ridgefield, NJ, USA); Gel-One^®^ (Zimmer, Seigakaku Corporation, Cambridge, MA, USA); Hyalubrix^®^ (Fidia Farmaceutici, Spa-Abano Terme (PD), Italy); Supartz FX™(Bioventus, Durham, NC, USA); Orthovisc^®^ (Anika Therapeutics, Bedford, MA, USA); Euflexxa^®^, previously named Nuflexxa (Savient Pharmaceuticals, Inc., Bridgewater, NJ, USA); Monovisc^®^ (Anika Therapeutics, Bedford, MA, USA); and Gel-Syn™ (Institut Biochimique SA, Pambio-Noranco, Switzerland) [33]. Each product differs in many characteristics, including source (animal vs. bacterial biofermentation using modified organisms), mean molecular weight ranging from (500 to 6000 kDa), distribution of molecular weight, molecular structure (linear, cross-linked or both), method of crosslinking, concentration (0.8–30 mg/mL), volume of injection (0.5–6.0 mL), and posology [34], although animal source of HA (rooster combs) was considered as a traditional source for many years. The alternative ways of producing hyaluronic acid currently in use, such as bio-fermentation using genetically modified organisms, are the result of many investigations that have been carried out over time. This modified bacterial source is currently used as the main source as it is associated with lower costs and fewer side effects [35,36]. For oral HA treatment, the body absorbs the high-molecular-weight polymer as a decomposed 2–6 membered polysaccharide [37]. One proposed mechanism of action shows that ingested HA binds to toll-like receptor-4 and promotes the expressions of interleukin-10 and cytokine signaling, which both lead to anti-inflammation of arthritis [38]. In a systematic review of 13 reports on oral HA clinical trials, Oe et al. found that patients that were on a highly pure HA regiment reported a beneficial effect on knee pain compared to placebo [31]. In terms of safety, it has been shown that in a 12-month study of 30 patients taking an oral HA regiment, no statistically significant negative side effects were seen [39]. As stated before, locally injected HA is distinct in many different characteristics. The most fundamental change is the molecular weight of the HA in the injection, and it was shown that there is no significant difference in the long-term outcome regardless of the preparation [40]. Unlike oral treatment, the complete HA molecule is introduced to the synovial fluid of the affected joint, providing a variety of different mechanisms for symptom relief [41]. These include enhancing the synthesis of extracellular matrix proteins, altering inflammatory mediators in order to shift away from degradation, reducing the motility of lymphocytes, and maintaining cartilage thickness, area, and surface smoothness [32]. However, it must be stated that these are not the only proposed mechanisms of action for locally injected HA, and further research trials need to be performed in order to fully investigate the physiological effects of the treatment. Based on the study of 76 trials by Bellamy et al., locally injected HA treatment is an effective treatment for OA based on its effects on patients’ pain, function, and patient global assessment [42]. In terms of safety, it has also been shown that there are no statistically significant negative side effects in patients receiving injection treatment [43]. Studies have shown that both local injections and oral supplementation of HA can combat OA symptoms, especially with those with early osteoarthritis [44]. Interestingly, Panuccio et al. showed that if these two types of treatments are combined, the oral supplementation of HA can extend the benefits of the injection treatments [45]. Thus, patients would not have to visit hospitals and receive the sometimes uncomfortable injections as often [45]. Further randomized clinical trials are required to be designed in order to determine the exact outcomes of combined treatment. Many studies on OA of the hip indicated that the intra-articular use of HA products may be a relevant option in the management of patients suffering from hip OA with persistent pain, who do not respond to conventional analgesic or pharmacological treatment alternatives [39,40]. However, the evidence suggesting improvements with these injections is weak [41], and controversial results can be found in the literature on the clinical benefit of HA injections [38,42,43]. Indeed, Holen et al. in a systematic review observed a benefit of intra-articular HA inside joints including the hip but concluded that the benefit was not better than placebo [43]. The effectiveness of the procedure can be influenced by several factors. Therefore, it is important to carefully select the patients who will be offered this treatment to increase the success rate. In 2017, Eymard et al. [40], on behalf of the Osteoarthritis Group of the French Society of Rheumatology and the French Research Group in Interventional Rheumatology, published their results from a multi-center, open-label, prospective trial. They evaluated the subset of hip OA patients that benefited the most by a single intraarticular injection of a cross-linked HA combined with mannitol on day 90. Patients with moderate pain, moderate disability, moderate joint space narrowing, superomedial, and axial femoral head migration, with femoral–acetabular impingement or coxa profunda displaying better results. They reported a reduction of pain in 50% of their patients on day 90. Benefits in a similar subset of patients have been reported by other authors as well. Pogliacomi et al. [44] in their study published in 2018 reported the efficacy of intraarticular single dose of high-weight HA in patients under a follow-up of 12 months. They concluded that patients with a moderate grade of OA are the ones that benefit the most from the said injection, also in accordance with our study.

In our series, 21.2% of the patients had transient pain during the days immediately after the procedure. One of the problems that can be observed is the extravasation of HA that could cause inflammation of periarticular tissues and must be avoided, as it can sometimes mimic a septic reaction with pain. Acute local reactions have been associated with multiple injections, so they concluded that a single injection is more beneficial [45]. In a meta-analysis focused on adverse events after intra-articular HA, Wu et al. [46] could not find an increased adverse reaction rate with HA compared to saline controls. They showed that hyaluronic acid is more effective in the long-term, but corticosteroids are more effective than hyaluronic acid in the short-term [47].

In 15.3% of the patients, symptoms persisted despite the injections, and THA was needed. Retrospective analysis of THA rates in a large series on hip OA with HA injection suggested that injections are useful to postpone arthroplasty [48]. However, HA does not seem effective in severe hip OA. In advanced stages, the treatment could be considered only in patients who refuse a THA. These patients must be informed that the clinical benefit could be poor. Moreover, in the event of a lack of clinical benefit from injections, THA must be delayed for a minimum of 3 months due to the reported increased risk of periprosthetic joint infection in the preceding period, as different authors suggested [49,50]. More generally, the effectiveness of this procedure is still debated for effective and durable benefits. To clarify some aspects of viscosupplementation treatment, a review of the literature confirmed that IA HA is an effective treatment for mild to moderate osteoarthritis but it is not an alternative to surgery in advanced cartilage degeneration [51]. Another important aspect is the technique that you can use to perform injections. Giordano et al. concluded that use of an automated delivery system for US-guided intra-articular hip injections did not show superior efficiency or patient comfort over traditional ultrasound-guided syringe injections [52]. In our daily practice, we do not use these systems both because the US guidance alone is deemed sufficient for the success of the procedure and because there is more margin of maneuver of the probe and the needle independent of each other.

This study has some limitations. The absence of a control group did not allow us to compare the efficacy of UGIs with HA with other products, including steroids, platelet-rich plasma, and other orthobiologics. Although we monitored which patients needed to undergo THA, the follow-up was not long enough to draw conclusions on the medium- and long-term efficacy of this treatment or whether repeated treatment over time would have the same efficacy. We did not include instrumental investigations to monitor the radiographic progression of the arthrosis or an MRI in the younger patients to assess the effect on the chondropathy. Therefore, conclusions are limited to the clinical side only.

## 7. Conclusions

Based on the results of this study, we can recommend the use of high-molecular-weight HA UGIs in patients with mild to moderate hip OA (Tonnis 0–2). Further studies are necessary to understand the predictors of response, the diversity of response to different HA products, and the appropriate dosage and timing in relation to the severity of the disease. Our study supports the use of high molecular weight HA UGIs in patients with mild to moderate hip OA (Tonnis 0–2) to obtain pain relief and clinical improvement up to 1 year of follow up. It is a treatment with few side effects and is repeatable over time, with the potential to delay the need for hip replacement in combination with interventions on other risk factors. Further studies are necessary to understand the predictors of response, the diversity of response to different HA products, and the appropriate dosage and timing in relation to the severity of the disease.

## Figures and Tables

**Table 1:** Patients divided according to Tonnis’s Classification for OA.

Tonnis Grade	No. of Patients
0	20
1	32
2	18
3	15

**Table 2:** Correlation between Tonnis’s Grade and patients reported outcomes before and 12 months after HI.

Tonnis Grade	mHHS (Pre-Post)	HOS (Pre-Post)	HOSs (Pre-Post)	WOMAC (Pre-Post)	VAS (Pre-Post)
0	56–91	79–82	50–69	24–7	7–1
1	60–63	65–73	37–43	36–30	6–5
2	59–64	70–72	49–51	32–33	6–5
3	60–62	70–71	47–50	33–35	6–5

## Data Availability

The data presented in this study are available on request from the corresponding author.
